# Usability challenges with electronic health records (EHRs) during prerounding on pediatric inpatients

**DOI:** 10.1093/jamiaopen/ooac018

**Published:** 2022-03-08

**Authors:** Jawad Alami, Clare Hammonds, Erin Hensien, Jenan Khraibani, Stephen Borowitz, Martha Hellems, Sara Lu Riggs

**Affiliations:** 1 Department of Engineering Systems and Environment, University of Virginia, Charlottesville, Virginia, USA; 2 Department of Computer and Communication Engineering, American University of Beirut, Beirut, Lebanon; 3 Department of Pediatrics, University of Virginia, Charlottesville, Virginia, USA

**Keywords:** EHR, pediatric, usability, prerounding

## Abstract

**Objective:**

Prerounding is critical for a healthcare team to develop a shared understanding of the patient’s condition and to develop a care plan. However, the design of electronic health records (EHRs) often makes prerounding inefficient, ineffective, and time consuming. The goal of this study was to observe how residents use the EHR while prerounding to identify usability challenges associated with the design of EHRs.

**Materials and Methods:**

Thirty residents were tasked to preround 2 pediatric patients using the think-aloud protocol. The data from the surveys, video recordings, and think-aloud comments were analyzed to identify usability issues related to EHR. The time it took for participants to complete the 6 required prerounding tasks were calculated and the pages most commonly accessed were noted.

**Results:**

Participants spent on average 6.5 min prerounding each patient with the most time spent on checking lab results and reviewing notes. Twenty-eight distinct pages were visited by at least 2 participants, mostly due to a lack of interconnectivity between related data across pages. Usability issues with the most commonly used pages include: data overload, missing/hidden information, difficulty identifying trends, and having to conduct manual calculations.

**Conclusions:**

We list usability issues and provide a set of recommendations to remedy these issues that include: reducing information access cost, creating a checklist, automate calculations, and standardizing notes and EHR training. Ideally, the outcome of this work will help improve EHR design to maximize the time clinicians spend interacting with and providing care to their patients.

## Lay Summary

Residents spend a significant amount of time using the electronic health record (EHR) system and these interactions can often be frustrating, inefficient, and time consuming. This study focuses on identifying challenges associated with EHR use during prerounding by residents. As part of this study, we observed 30 residents while they preround 2 pediatric care patients using the EHR system. The results of our study showed that the design of EHRs currently do not support the tasks the residents need to perform while prerounding. Patient data are spread across numerous pages within the EHR system and the information is often not displayed in the most user friendly manner. This adversely affects the effectiveness and efficiency of prerounding. This study provides a set of recommendations to support prerounding that include dashboard design guidelines, standardizing data entry, and automating certain tasks within the EHR.

## INTRODUCTION

Rounding is a critical process for patient care in inpatient hospital units. The primary purpose of inpatient rounds is for the entire healthcare team to quickly develop a shared understanding of the patient’s condition so that the team can collaboratively formulate an assessment of the patient’s condition and develop a care plan. In teaching hospitals, resident physicians typically gather and compile information on their assigned patients prior to the start of the rounds, in a process referred to as *prerounding*.^1^ Through prerounding, a resident creates a mental model about each patient’s current condition, which is then conveyed to the healthcare team to decide on the best course of action. As such, this makes prerounding a critical aspect in clinician decision making.

Most of the information residents collect during the prerounding process comes from the electronic health record (EHR) system. The workday may begin with the handoff of patients’ care to interns and residents on the day team from the overnight team.^2^ Residents are tasked to collect information from the EHR system, organize it in an appropriate manner, and present this information to the patient healthcare team during rounds. However, a major challenge is the enormous amount of data the residents need to consider.^3^ For example, a study performed more than a decade ago on ICU patients concluded that an average of 1348 data items about the patient are added to the EHR each day.^4^

Given that prerounding needs to be completed in timely manner, many of the challenges associated with prerounding stem from the EHR system usability issues.^5^^,^^6^ Key pieces of information needed for prerounding are often located in different sections of the EHR, requiring residents to have a priori knowledge about where the information is located and how to navigate and find this information.^7^ This challenge is compounded by the fact that residents are receiving inconsistent and often inadequate training in using the EHR system. Previous work has shown that clinicians working with a well-established EHR still omitted 32% of lab data from ICU rounds’ presentations.^8^ Interventions to facilitate prerounding in the EHR have been shown to improve the workflow (eg, automated EHR-generated rounding report^9^).

The goal of this study was to observe how residents use the EHR while prerounding to identify usability challenges associated with the design of the system. This initial study is the first step in streamlining the prerounding process at the University of Virginia, and in developing general EHR design guidelines. Ideally the outcome of this work will help decrease the amount of time clinicians spend performing tasks associated with the EHR to increase the time clinicians spend interacting with their patients and providing care to their patients.

## METHODS

### Participants

Thirty pediatric residents at the University of Virginia participated in this quality improvement project. The residents’ training ranged from 1 to 3 years of postgraduate medical education (PGY1–PGY3), with a median of 2 years of experience. Prior to participating in the study, all participants had experience using the EHR system (Epic Systems**^®^)** to complete the tasks associated with prerounding while on the job. The institutional review board at the University of Virginia approved this study and all participants gave informed consent prior to the start of the study.

### Experimental setup

Residents simulated prerounding in an experimental setting as part of an optional professional development event that was available to all pediatric residents. Each participant was provided a 17.3″ Lenovo workstation laptop and a wireless mouse to perform their usual prerounding tasks on 2 different complex pediatric inpatients. During the study, participants were near each other, and the noise generated during the study simulated the environment in which prerounding typically occurs. Participants could take notes using paper and pen/pencil. For the data collection, the Morae^®^ video analysis software (TechSmith, USA) was used to capture audio, video, user inputs (mouse movements and clicks), and on-screen activity.

### Tasks

Prior to the experimental portion of the study, participants completed a web-based questionnaire that included 1 demographic question and 6 pertaining to EHR usability and the prerounding process. On the day of the study, participants completed a second questionnaire that included additional questions regarding EHR use.

During the study, participants were asked to preround 2 actual patients. The patient cases were chosen to resemble routine acute-care pediatric inpatient cases that are typically assigned to the residents. The participants were asked to log into the EHR system on the provided laptop, start the usability software, then use the EHR system to preround on their assigned patients (*Note: User customizations and preferences were automatically loaded when the participants signed into the EHR system*). Participants were instructed to use the think-aloud protocol, that is, verbalize what they were thinking while completing the tasks. Participants were assigned to 1 of 2 groups of 15 residents. The order in which the patients were assigned to the residents to preround was randomized. Each participant was given 20 min to complete the prerounding task.

After prerounding on both patients, participants were instructed to stop the usability software from recording. A debriefing questionnaire then popped up and asked the participants 3 questions regarding issues faced when completing the prerounding scenario such as time limit concerns and difficulty finding certain information.

### Video/on-screen activity analysis

The video/on-screen activity recordings for each participant were analyzed by a team that included 3 undergraduate researchers, 1 graduate researcher, and 1 academic advisor who are well-versed in the process and outcomes expected at the conclusion of the prerounding process. The data were analyzed using the following 4 steps:

#### Step 1: Identifying events to be coded in the video analysis

The residents were expected—at the very least—to complete the following during prerounding: (a) review the flow chart of the patient, (b) note major events that occurred over the past day, and (c) track down events that occurred overnight.^10^ According to pediatric experts, residents perform the following 6 subtasks when prerounding:

- Review vital signs (Vitals),
- Check feeding and lab orders (Orders),
- Review lab results (Labs),
- Check intakes and outputs (I/Os),
- Review notes (Notes), and
- Review current medications, dosages, and medication changes (Meds).

For the video analysis we flagged and coded the videos to identify the following 5 events to be used for data analysis:

1. *Start/end of prerounding a patient.* How long it took to preround each patient.
2. *Start/end of a subtask.* How long it took each participant to complete the subtasks of interest (ie, A–F above).
3. *Page access.* When participants navigated from one page to another.
4. *Information/data collection.* When and where each participant collected a particular data piece from the page they were viewing.
5. *Participant comments.* When participants commented on usability issues that they encountered in real-time while using the EHR system using the think-aloud protocol.

#### Step 2: Coding videos for events

A spreadsheet template was used to record when the 5 events from step 1 occurred in the video recordings. Each reviewer would note all applicable information that were relevant to the event (ie, event type, prerounding task, subtask, and page). Multiple passes over different segments of each video were often performed by reviewers to fully capture all details.

#### Step 3: Data validation and consolidation

Each video recording was initially reviewed by 2 reviewers separately. A third reviewer would then compare the 2 resulting spreadsheets and identify any discrepancies. When a discrepancy was found, the third reviewer would refer to the video recording again to determine how to best consolidate the 2 streams of data. Typically, there was consensus for all events across all videos, with the timings occasionally being off by a few seconds.

#### Step 4: Data reduction

After consolidating the dataset, a final reviewing pass was done by one of the reviewers, which included merging entries that were relatively similar, and creating a second version of the dataset, which was better suited for analysis. All proposed changes or modifications were agreed upon by all reviewers before moving onto the analysis portion of the study.

## RESULTS

The results below summarize key information about how the participants spent their time prerounding and usability issues they encountered. We were only able to analyze video recordings of the prerounding process from 20 participants (16 female, 4 males) due to video data quality of some participants.

### Time spent on each subtask

**Figure 1.**
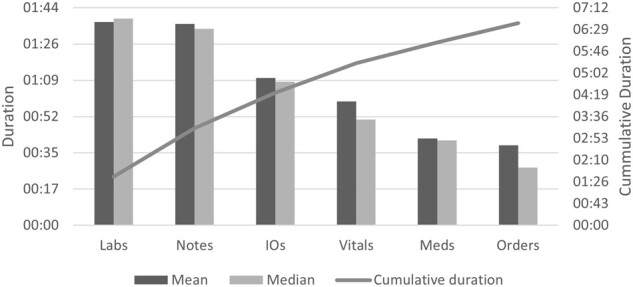
Mean and median time in minutes across participants spent on each of the 6 subtasks.

Participants spent on average 6:27 min prerounding each patient. Figure 1 shows that almost 50% of the prerounding time was spent on “Labs” (ie, checking lab results) and “Notes” (ie, reviewing patient notes). Routine tasks of checking and reporting “I/Os” and “Vitals” (patient vital signs) made up about 33% of the prerounding time. The remaining 17% of the prerounding was spent on reviewing “Meds” (ie, medications) and checking patient “Orders”.

### Number of pages accessed for each subtask

In the web-based questionnaire, participants were tasked to complete prior to the experimental session, roughly 70% of participants indicated that they found some information hard to access within the EHR and 33% commented that information is inconveniently spread out throughout the EHR system. These responses were consistent with the results from the video analysis that showed that more than 58 different pages were visited by the participants while performing the prerounding task for the 2 patients.

**Figure 2.**
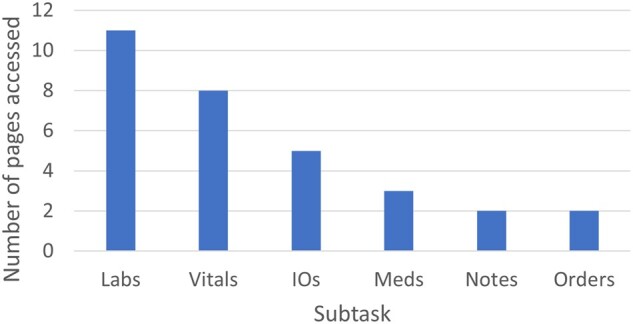
Number of pages accessed by participants for each subtask.

The mean number of pages accessed for each participant was 12.3 pages per patient (median = 12 pages). While this number is certainly large, it is only around one-third of the total pages accessed by all participants. Figure 2 shows the number of pages accessed by the participants for each subtask for both patients with the number of pages accessed ranging from 2 to 11 page depending on the subtask. Data were most spread out for the “Labs” and “Vitals” subtasks, moderately spread out for the “I/Os” and “Meds” subtasks, and least spread out for the “Notes” and “Orders” subtasks.

We only included the 28 pages that were visited by at least 2 participants in these counts as we assumed that pages accessed by a single participant were not typically used by residents during prerounding, and thus were excluded from the analysis. Three pages were accessed for multiple subtasks and were included in the counts for each of these subtasks; these pages include similar information with varying levels of detail or different forms of representation (eg, timelines, tables, graphs, ranges, and text). For example, the “Ped Rounding” page under the summary tab includes summarized information related to “Vitals”, “I/Os”, and Patient History; however, the Intake/Output information could also be found in the “Flowsheets”, “Manage Orders”, “Summary I/Os”, and the dedicated “Intake/Output” page. While all mentioned pages include similar information, the form of representation and level of detail varied greatly between pages.

Currently the different pages and sources of information lack interconnectivity. This was noted by the participants’ responses to the questionnaire, verbal comments during the prerounding task, and our observations when reviewing the video data. Pages that provide aggregate summaries of information often do not provide a mechanism for the user to view further details about the aggregated information and how it was derived. For example, if a user is viewing the “Ped Rounding” page to collect intake and output information and would like to get more details about the type(s) of intake that the patient received, there are currently no links between this page and the corresponding pages that list intake types. Instead, the user must manually navigate to the “Flowsheets” page, navigate to the “Peds I/O” subpage, select an option to view the intake, then scroll and search for the different intake events. This process is described by our participants and video data reviewers to be tedious, time consuming, and disorienting.

### Time spent on pages

The top 4 pages the users spent the most time on include: “Notes”, “Results Review”, “Intake/Output”, and “Pediatric Overview”. To calculate the time spent on each page by each participant, we summed the duration of all instances the page was viewed, excluding the time spent on pages that were accessed by mistake, accessed for only a few seconds, had no data extracted for the purpose of prerounding, or accessed to mainly to access another page (ie, to gain access to another subpage within it). We omitted from our analysis pages that were only accessed by one participant as they are likely not used in the typical workflow. For each page, we have noted usability issues that participants reported in their questionnaire responses or while performing the prerounding task in addition to reviewers’ own observations which will now be discussed in turn.

*Notes:* Video analysis showed that collecting data from the “Notes” page was the most time consuming. Although the mean time spent on the page was 1:28 min, some participants spent almost half of their time reviewing notes. Based on our observations, physician notes were often lengthy and were “bloated” with information that went beyond the intended scope of the note and/or with information readily available elsewhere. Participants had to skim through lengthy paragraphs to collect relevant information as note summaries were not available and there was a lack in consistency in text formatting.

*Results* *review* *:* This included all lab and imaging results. Although the “Results Review” page alerted the user to out-of-range results, residents had to hover over the results to see how the values compared to normal ranges. The main “Results Review” page ideally includes a summary table of *all* test results; however, this was not always the case. Residents sometimes had to navigate to other subpages to access the results. This meant that unless a resident checked the “Lab Orders” or the “Notes” page, they might be unaware of certain tests and results that are not included in the summary table. Additionally, the current design makes assessing trends tedious and time consuming.

*Intake/output:* Participants spent a significant amount of time on this page even though it mainly listed intake/output types and corresponding volumes. Residents cited they had to do manual calculations for the intake volume based on the patient’s weight. Although this functionality is available in the EHR, the values are currently not calculated by the system in a timely manner for a myriad of reasons (eg, incomplete data, data not entered by the responsible party, etc.). Residents also noted that more detailed information about intakes and outputs are not accessible from this page and require them to visit other pages to get the data. We saw in our observations that certain information was hidden/collapsed by default (eg, emesis events), so a user might overlook these events unless they expanded all rows.

*Pediatric* *overview* *:* This page included all raw data related to ‘Vitals’, ‘Labs’, and ‘I/Os’. The data are presented in a large table sorted by time, and residents often used the page to check the co-occurrence of events. There is a large amount of data on this page/report, and it does not afford an alternative mechanism to compare values and visualize trends.

Table 1 summarizes the pages that had the highest average access duration, average duration of time spent on each page, the associated subtask(s) that the page serves, and the page’s corresponding usability issues.

**Table 1.** Summary of the duration spent on each page, subtasks associated with each page, and usability issues associated with each page

Page	Duration (min)	Subtask(s)	Usability issues
Notes	1:28	Notes	Note bloat Easy to miss important information
Results review	1:06	Labs	Missing information Disorienting scrolling Hard to see trends Hover to access information
Intake/output	0:56	I/Os	Manual calculations Hidden data No option to get more details within the page
Summary/pediatric overview	0:44	Vital Labs I/Os	Raw data Data overload Easy to miss trends

## DISCUSSION

Our goal was to understand how residents use an EHR system while prerounding and identify usability issues. The findings show that the current state of the EHR system is not well suited for their workflow needs. Our study has found numerous usability issues with regard to the design of the pages that hampers residents in performing the prerounding task. We list these usability issues and provide a set of recommendations to remedy these issues:

- *Reduce information access cost*. We saw that the location of data and varying levels of specificity of data were often spread throughout the EHR system, and the information was not linked. Navigating between pages to access different information has been found to be disruptive to the residents’ workflow^11^ and increase cognitive workload.^12^ At the hospital in which this study was conducted, there have been previous efforts to create dashboards that aggregates data from different pages within the system to support prerounding (ie, “Pediatric Rounding Report” page). However, during our observations we saw only 2 residents use this page. This suggests that either the dashboard does not support the residents’ information needs during prerounding or residents are unaware of its existence. The 2 participants who used this dashboard accessed slightly fewer pages on average compared to the other participants, but spent a longer time on prerounding (mean = 8:02 min). The longer prerounding times by these participants may suggest that the dashboard design was not optimized to reduce prerounding time. We believe that the concept of a dashboard that localizes relevant data is promising but recommend that its design—and the design of all pages within the EHR system—be modified and iteratively improved to better support the users. To this end, based on our findings we recommend a dashboard that (1) includes better data visualizations to identify patient trends and (2) better links relevant data to minimize information access costs. This study is also valuable to identify how users are using the system to identify the disconnect between what the users need compared to what they want.
- *Create a prerounding checklist*. Researchers have found that clinicians working with a well-established EHR system still omitted 32% of lab results during ICU rounds presentations.^8^ A possible contributor to these omissions based on our observations and participants comments during the think-aloud protocol is (1) missing information from the main lab results page (ie, “Results Review” page that includes all patient test results) and (2) disorientation that occurs when scrolling through large chunks of text and data. Previous work has shown that supporting knowledge in the world versus head—that is, reduce recall and memory—is a more effective mechanism to address omissions.^13^ To minimize the likelihood of omitting information collected during prerounding, we recommend the use of checklists that include prompts (eg, fill in blanks) that remind residents of what information is needed instead of relying on the residents’ memory each time they preround. Other approaches could include creating rounding tools that help the entire team visualize the patient’s status during rounds and/or distributing the work so that somebody on rounds is responsible for “looking up” and reporting desired data elements.
- *Automate calculations*. Physician notes, orders, and lab results vary greatly between patient cases as each case has its own diagnosis, clinical trajectory, and course of treatment. It was expected that residents would spend most of their time on these subtasks; however, almost a third of the prerounding time was spent on collecting information on “I/Os” and “Vitals”. Ideally, collecting information about “I/Os” and “Vitals” meant copying the information on the screen onto paper, but residents spent a lot of time doing manual calculations and navigating multiple pages to find this information. These aspects related to “I/Os” and “Vitals” contributed to a lot of the frustrations cited by residents in their open-ended responses in the questionnaire and during the verbal portion of the think-aloud protocol as residents spent more time on these tasks than they wanted. We recommend that there be a mechanism in the EHR system to offload these manual calculations that currently need to be done by hand by the resident. However, given the importance of these calculations in patient care, we also recommend having a mechanism to double check these calculations by having all the pertinent information on the same page showing how the calculation was derived and having an embedded calculator within the system.
- *Standardize notes*. Previous studies have shown that note bloat—that is, unnecessarily lengthy notes—increases the difficulty and the time it takes residents to extract relevant information related to patient care.^14^ Our observations also showed that reviewing notes was the most time-consuming task even though the spread of these data was relatively minimal compared to the other evaluated subtasks. We saw that the lack of standardization made it difficult to find information. As such, we recommend a standard way for inputting notes such as the Assessment, Plan, Subjective, Objective (APSO) note format.^14^ Standardizing notes could mitigate some of the effects related to “note bloat” as important information would be easier to locate. This will ideally provide a springboard for the clinician to assess a patient’s state that goes beyond information extracted from the EHR, addressing questions that include: *what* is the patient’s status (ie, whether the condition has improved or deteriorated over time), *why* the patient is trending in a certain direction, and *what* are his/her plans for the patient.
- *Standardize prerounding*. On 2 separate occasions we saw participants using nonstandard procedures to perform their prerounding task and gather data. Instead of visiting individual pages to collect patient data, these participants used a workaround of creating a new note within the system, then used shortcuts (ie, Dot-Phrases) to populate the new note with vital ranges, I/O data, and recent lab results. The users then deleted the new note after collecting the data provided by the Dot-Phrases. This workaround helped users cut down on the time needed to collect these data by not having to visit multiple pages within the system.

Additionally, multiple participants indicated that the system does not provide means to quickly comprehend the patient’s current situation or why certain actions were taken by the medical team. Instead, they rely on conversations with nurses to get briefed on a patient to help them make sense of the data in the system.

The use of such workarounds highlights some shortcomings of the user interface and a flaw in integrating the technology within users’ workflow. While these workarounds are saving residents time and energy, the savings might be at the expense of the safety of the patient and the quality of their care. By relying on oral communications, residents are at the risk of missing or omitting important information that might be critical to the patient care in the EHR; similarly, the use of shortcuts within notes to gather information might be prone to errors as such functionalities are designed for documentation purposes and not for gathering information. This emphasizes the urgency of redesigning the system in a way that better suits the needs of users, as the current state is jeopardizing the safety and quality of patient care.

### Limitations

Our prerounding simulation was conducted with only pediatric residents and patients, which may limit generalizability to other specialties. Similarly, this study was performed in one health system using one implementation of Epic. While Epic is widely used in the United States, the results may vary for other EHR systems and other implementations of Epic. However, many of the usability findings—for example, minimizing access cost—are universal in nature. Participants were asked to use the think-aloud protocol, which might have been unnatural and distracting while performing their tasks. However, the information gained from the think-aloud protocol as part of this study justified its use to shed light on the mental models adopted by the participants and the challenges associated with completing the prerounding task.

## AUTHOR CONTRIBUTIONS

JA, SLR, and SB conceived and planned the study. JA, SLR, SB, and MH conducted the study and collected the data. JA, CH, EH, and JK took the lead in extracting, cleaning, and analyzing the data. SLR, SB, and MH also contributed to the data analysis. JA, CH, EH, JK, SB, MH, and SLR wrote the manuscript.

## CONFLICT OF INTEREST STATEMENT

None declared.

## DATA AVAILABILITY

The data underlying this article cannot be shared publicly to protect the privacy of individuals that participated in the study. However, the data will be shared on reasonable request to the corresponding author.
